# How to do (or not to do)…using causal loop diagrams for health system research in low and middle-income settings

**DOI:** 10.1093/heapol/czac064

**Published:** 2022-08-03

**Authors:** Rachel Cassidy, Josephine Borghi, Agnes Rwashana Semwanga, Peter Binyaruka, Neha S Singh, Karl Blanchet

**Affiliations:** Department of Global Health and Development, London School of Hygiene and Tropical Medicine, 15-17, Tavistock Place, London WC1H 9SH, UK; Department of Global Health and Development, London School of Hygiene and Tropical Medicine, 15-17, Tavistock Place, London WC1H 9SH, UK; Information Systems Department, College of Computing and Information Sciences, Makerere University, PO Box 7062, Kampala, Uganda; Ifakara Health Institute, PO Box 78373, Dar Es Salaam, Tanzania; Department of Global Health and Development, London School of Hygiene and Tropical Medicine, 15-17, Tavistock Place, London WC1H 9SH, UK; Geneva Centre of Humanitarian Studies, University of Geneva and the Graduate Institute, 40, Boulevard du Pont-d’Arve, 1211 Genève, Switzerland

**Keywords:** Health systems research, research methods, study design, complex systems, systems thinking, causal loop diagrams, low- and middle-income countries

## Abstract

Causal loop diagrams (CLDs) are a systems thinking method that can be used to visualize and unpack complex health system behaviour. They can be employed prospectively or retrospectively to identify the mechanisms and consequences of policies or interventions designed to strengthen health systems and inform discussion with policymakers and stakeholders on actions that may alleviate sub-optimal outcomes. Whilst the use of CLDs in health systems research has generally increased, there is still limited use in low- and middle-income settings. In addition to their suitability for evaluating complex systems, CLDs can be developed where opportunities for primary data collection may be limited (such as in humanitarian or conflict settings) and instead be formulated using secondary data, published or grey literature, health surveys/reports and policy documents. The purpose of this paper is to provide a step-by-step guide for designing a health system research study that uses CLDs as their chosen research method, with particular attention to issues of relevance to research in low- and middle-income countries (LMICs). The guidance draws on examples from the LMIC literature and authors’ own experience of using CLDs in this research area. This paper guides researchers in addressing the following four questions in the study design process; (1) What is the scope of this research? (2) What data do I need to collect or source? (3) What is my chosen method for CLD development? (4) How will I validate the CLD? In providing supporting information to readers on avenues for addressing these key design questions, authors hope to promote CLDs for wider use by health system researchers working in LMICs.

Key messages

- Causal loop diagrams enable identification and visualization of drivers for complex system behaviour, including spill over effects or unintended consequences of policy and managerial decisions.
- They can be built and validated using different sources of data, depending on resource and health system setting constraints.
- It is vital that further research using a systems thinking lens be conducted in LMICs, taking into account the delivery and resource constraints experienced by facilities and actors, to further our understanding of health system functioning and optimization.

## Introduction

Health systems are complex systems due to the large number of system elements (people, resources, processes), the varying and extensive relationships between them, and their responsiveness to their external environment (Lipsitz, 2012; Barasa *et al.*, 2017). They produce non-linear behaviour that evolves over time (Sterman, 2000a) and in response to relationships that exist between system elements (Lipsitz, 2012). Treating the health system as a static, linear system in evaluations results in oversight of potential unintended consequences, with health policies leading to suboptimal or undesirable outcomes due to focus on singular events and failure to observe the feedback and relationships between system elements (Adam and de Savigny, 2012).

For this reason, tools designed to manage and analyse complex behaviour need to be used to guide the design of health system interventions, and evaluate their effects (Skivington *et al.*, 2021). In taking a ‘systems thinking’ approach to research, emphasis is placed on connections and relationships between system elements as part of a larger, evolving system (Peters, 2014). Methods derived from systems thinking enable evaluation of interventions on the wider, interconnected dynamic system whilst observing the important underlying mechanisms and interactions that drive health system behaviour (Gates, 2016). Causal loop diagrams (CLDs) are one such method providing a visual representation of the relationships between system elements and their interactions, leading to understanding of what drives problematic system behaviour (Adam, 2014).

By helping to identify key health system constraints and/or evaluate potential health system improvements prior to implementation to guide programme design, CLDs can ensure investments are well targeted, which is especially useful in resource constrained health systems Furthermore, CLDs can be employed even where routine health information system data are limited, as literature, policy reports and stakeholder interviews can be used to support development of models. CLDs can be used to better understand the ‘mechanisms for action’ in the health system before interventions are implemented to inform their design (Borghi and Chalabi, 2017), or after their implementation to determine what worked, how and why.

However, to date, the use of CLDs has been limited in health systems research in low- and middle-income country (LMIC) studies (Borghi and Chalabi, 2017; Cassidy *et al.*, 2019). This paper introduces the reader to CLDs and their potential usages as a health systems research and policy tool, with particular attention to issues of relevance for LMIC studies. We then guide the reader through the stages of CLD development and validation (Box 1), using examples from the LMIC literature and authors’ own experience of using CLDs in Tanzania and Uganda.

Box 1. **Four guiding steps that underpin the design and conduct of CLDs for health systems research**

**(1) What is the scope of this research?**

To define the phenomena or behaviour that you are trying to unpack, there are three key elements to consider:

- Time frame of interest
- Boundary of issue
- Level of system aggregation

**(2) What data do I need to collect or source?**

To further understanding on what is driving phenomena/behaviour, we can source and analyse:

- Primary data (e.g. key informant interviews and group model building)
- Secondary data (e.g. programme evaluation data, published literature, health surveys or reports, policy documents and systematic or realist review).
- Primary and secondary data

**(3) What is my chosen method for CLD development?**

Method for analysing and extracting data for CLD development:

- Ex post development (e.g. thematic analysis and purposive text analysis)
- Real-time development (e.g. group model building)

**(4) How will I validate the CLD?**

Method for confirming the CLD is still grounded in the experience of those with expert knowledge of the phenomena/behaviour:

- Stakeholder dialogue, including group model building activities
- Comparison to primary/secondary data sources

### What are CLDs?

CLDs (Box 2) are diagrams that help us better understand what actions or mechanisms drive behaviour in a system (Tomoaia-Cotisel *et al.*, 2017); feedback (interactions between system elements, causing cycles of cause-and-effect behaviour) and loops (cycles of behaviour) emerge through development of these diagrams, illuminating desirable or undesirable behaviour (Sterman, 2000a). We can also identify spill-over effects of actions or interventions to wider parts of the system and unintended consequences that can lead to unexpected outcomes.

Box 2. **Origin, building blocks and interpretation of CLDs**

System dynamics (the methodological field in which CLDs originate from) began as a tool for industrial and business management but now has widespread application across various research domains, including health system research (Pruyt 2017).

**Figure:**
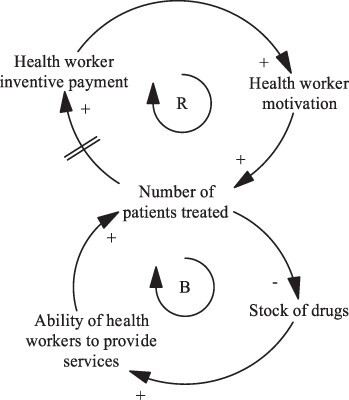
Notes to Box: Building blocks of CLD are presented; variables, arrows with polarity, reinforcing and balancing feedback loops and delays. Source: Adapted from Cassidy *et al.* (2021).

A simple CLD is presented in this box, showing the impact of a payment for performance intervention on the delivery of services at a health facility (Cassidy *et al.* 2021). Arrows with polarity indicate a causal relationship between two system variables and the direction of effect, for example, an increase in incentive payments during the intervention results in health workers feeling further motivated to deliver incentivised health services.

Delays in effect can also be represented, identified as a double line through an arrow. For example, we observe a delay in effect between the number of patients treated and an increase in the incentive payment then issued to health workers.

A series of arrows that close to form a ‘loop’ are labelled as either a reinforcing or balancing loop. A reinforcing loop exhibits amplified or spiralling behaviour (all arrows in the loop have the same polarity). An increase in health worker incentive payments leading to an increase in health worker motivation and the number of patients who are then treated leading to an increase in the incentive payments then issued to health workers is an example of a loop that shows reinforcing behaviour. A balancing loop is prevented from exhibiting spiralling behaviour by the presence of one or more variables and instead presents a dampened behaviour. An increase in the stock of drugs available at facilities results in an increase in health worker ability to provide services and the number of patients who are then treated. However, an increase in the number of patients treated results in a decrease in the stock of drugs at the facility.

For more information on interpretation and best practice for drawing CLDs (naming variables, identification of loops, etc.), please see Sterman (2000c) and Tomoaia-Cotisel *et al.* (2017)

### When can I use a CLD?

There are a variety of potential applications of CLDs of relevance to the health systems research and policy community. CLDs can be used ex-ante, to inform the design of a health systems intervention or policy, or to develop a theory of change to guide its evaluation (McGill *et al.*, 2021). Used in this way CLDs can determine the likely risks to a future programme that can be monitored during implementation to enable course correction (Sarriot *et al.*, 2015) and/or understand underlying mechanisms (drivers) for health system behaviour, and leverage points which can be targeted to produce optimum system behaviour (Kwamie *et al.*, 2014; Cassidy *et al.*, 2021). CLDs can also be used retrospectively to explore how policy implementation changes over time (Nigenda *et al.*, 2015), or to explore why health policies have succeeded or failed (Agyepong *et al.*, 2012; Paina *et al.*, 2014). They can be used in conjunction with existing health system frameworks, for example by identifying interconnections and/or dynamic behaviour between the WHO health system building blocks (Sharma *et al.*, 2020). Finally, CLDs can also support the synthesis of evidence regarding a health systems intervention, used to present the results of realist and systematic reviews (Namatovu and Semwanga, 2020; Singh *et al.*, 2021).

CLDs can also be used outside programme evaluation to explore how health systems respond to shocks or disruption (Ozawa *et al.*, 2016; Jamal *et al.*, 2020), and identify factors leading to system resilience, specifically the ‘absorptive, adaptive and transformative capabilities’ of the system. CLDs can highlight supply and/or demand side mechanisms related to a particular health condition, such as drivers for inadequate childhood immunization (Rwashana *et al.*, 2009; Varghese *et al.*, 2014; Kanniyan *et al*., 2021), uptake and provision of mental health services (Trani *et al.*, 2016; Noubani *et al.*, 2020) and refugee and host community demand for healthcare (Noubani *et al.*, 2020; Zablith *et al.*, 2021).

## How to design a causal loop diagram study for a LMIC health system setting

### What is the scope of this research?

When defining the scope of the CLD, there are three elements that need to be considered: the time frame of interest, the boundary of the issue, and the level of system aggregation (Kim, 2000). For what period of time did the policy or behaviour of interest unfold and therefore what period of time will be reflected in the CLD? What is the boundary i.e. where do we draw the line for what should be included in the diagram and what is external to it? Will the focus be on capturing community and/or facility dynamics (Rwashana *et al.*, 2014), or is the focus on district or state (Cassidy *et al.*, 2021), national (Paina *et al.*, 2014) and/or global level dynamics (Glenn *et al.*, 2020)? Relatedly, what is the level of aggregation in the CLD or level of detail needed to understand patterns of behaviour? To model the behaviour of interest, do actions and outcomes that occur on a daily, weekly, monthly or yearly basis need to be captured? When determining the scope of a CLD, the goal should always be to use CLDs to map key structural drivers for a given behaviour or problem of interest, not to try and map the feedback that drives behaviour in the entire, wider health system (Sterman, 2000a). This is key to avoiding overly complex diagrams which may obscure key dynamics around the behaviour or phenomena of interest.

The decision regarding the scope of the CLD can evolve during the process of the research, in response to discussion with stakeholders, new findings or resource availability for the project. In Cassidy *et al.* (2021), the research sought to determine constraints to achieving key service delivery targets in primary care facilities during a results-based financing programme. The time frame of interest was the duration of the programme (two years). The boundary and aggregation were informed by the research question (primary care facilities) and stakeholder experiences; their description of key events that led to their achievement or failure of targets during the intervention (at the facility, community and district-level) guided CLD development.

### What data do I need to collect or source?

CLDs can be generated using a variety of data sources, including primary and secondary data; often a combination of sources is used.

#### Primary data

Popular primary data sources include key informant interviews (Sharma *et al.*, 2020) and group model building (GMB) sessions (Noubani *et al.*, 2020). In GMB, development of the CLD takes place with direct real-time input from stakeholders present (more on GMB in the next section) whereas with key informant interviews CLDs are developed post-hoc. The purpose of data collection is to obtain causal information on drivers for a behaviour/phenomenon of interest; this information will then be mapped out in the CLD. Stakeholders can also be asked to comment on potential leverage points within the system and actions that could be taken to alleviate problematic behaviour which can be represented in the CLD.

A recent paper has compared CLDs developed from key informant interviews to those developed through GMB (Valcourt *et al.*, 2020). Although the CLDs developed from individual interviews yielded more variables and causal links, the CLDs produced from GMB workshops contained more feedback loops and more information on dynamic system behaviour. This was thought to be attributed to the design of GMB workshops, where stakeholders are actively encouraged to focus on feedback effects and dynamic behaviour. The decision to opt for key informant interviews versus GMB will be driven by several factors, including the availability of stakeholders, the topic under investigation (suitability for group discussion) and experience of the team. Due to global restrictions on travel during the recent COVID-19 pandemic, primary data collection has also successfully taken place through online mediums (Wilkerson *et al.*, 2020; Cassidy *et al.*, 2021).

Selection of stakeholders can be driven by researchers’ own knowledge of influential actors of the system under study or inferred from the literature. Who has expert knowledge of the problem we want to investigate? Those involved in funding, policy formulation, implementation and users/beneficiaries will have varying perspectives on the system and drivers for health system behaviour. Depending on the research question, different groups may need to be consulted to create a complete picture. Key informants can also be identified via snowballing during an initial round of interviews. Many studies in the current literature incorporate a provider perspective, with fewer including, or with the sole focus on the patient experience. Examples of study design and data collection tools for patients can be found in (Rwashana *et al.*, 2014; Zablith *et al.*, 2021).

#### Secondary data

Where a CLD is being used to understand causal pathways or programme mechanisms ex-post; programme evaluation data can be used to support the construction of a CLD (Varghese *et al.*, 2014; Sarriot *et al.*, 2015; Cassidy *et al.*, 2021). Other secondary data such as published or grey literature (Yu *et al.*, 2018; Kurnianingtyas *et al.*, 2020), health surveys or reports (Li *et al.*, 2019) and policy documents (Nigenda *et al.*, 2015) can also be used to develop CLDs. Data extracted through a systematic or realist review can be cleaned, integrated and categorized to generate cause and effect relationships that can be represented in a CLD (Namatovu and Semwanga, 2020; Singh *et al.*, 2021).

The decision to use secondary data to develop a CLD may be driven by difficulty in accessing stakeholders for primary data collection and/or a rich source of secondary data being available and suited for CLD development (Cassidy *et al.*, 2021). Whilst secondary data might be less resource intensive to obtain, care should be taken to ensure the data contributes causal information on what is driving behaviour in the system. CLDs developed using primary data can also be triangulated with evidence from the literature and other secondary sources (Alonge *et al.*, 2017; Ahmad *et al.*, 2019). For studies where repeated access to stakeholders for CLD development is not possible (e.g. humanitarian settings), a combination of primary and secondary data sources may be preferable.

The results from a CLD developed using secondary data can be presented to stakeholders for triangulation and validation to ensure key information has been retained in the diagram (Agyepong *et al.*, 2012; Cassidy *et al.*, 2021). Stakeholder engagement encourages buy-in to the research, with higher likelihood of uptake of findings by stakeholders and policy makers (Zimmerman *et al.*, 2016).

### What is my chosen method for CLD development?

There are different approaches for developing a CLD. Depending on the purpose of the research and data requirements, researchers may choose ex-post development (developing CLD from data collected/sourced) or real time development (developing the CLD with stakeholders). For further information on presentation of CLDs, see Box 3.

Box 3. **Tips for presentation of CLD**

**Software**

There are software packages specifically developed for creating CLDs such as Vensim (Ventana Systems Inc. 2015) and STELLA (Isee Systems Inc. 2021). Each have different licenses available to purchase depending on the functionality needed by the user. At the time of writing, Vensim offers a free personal learning edition for educational use.

**Presentation**

For ease of viewing, analysis and validation, it can be helpful to develop multiple CLDs or present the CLD in smaller segments (Sterman 2000b). Multiple CLDs can be used to demonstrate the policy effect and emergent behaviour (Paina *et al.* 2014; Renmans *et al.* 2017) and shifting community perspective on vaccine acceptance (Varghese *et al.* 2014) in the system at different time steps. A single CLD can also be presented in smaller segments, for example, key mechanisms related to the supply, demand and reporting of healthcare services (Rwashana *et al.* 2014; Cassidy *et al.* 2021); perception, causes and health seeking practices related to mental health (Noubani *et al.* 2020) can be presented in segments (that are connected in the wider, whole CLD). An example of how to illustrate where these segments connect in the wider CLD is given here (Cassidy *et al.* 2021).

**Figure:**
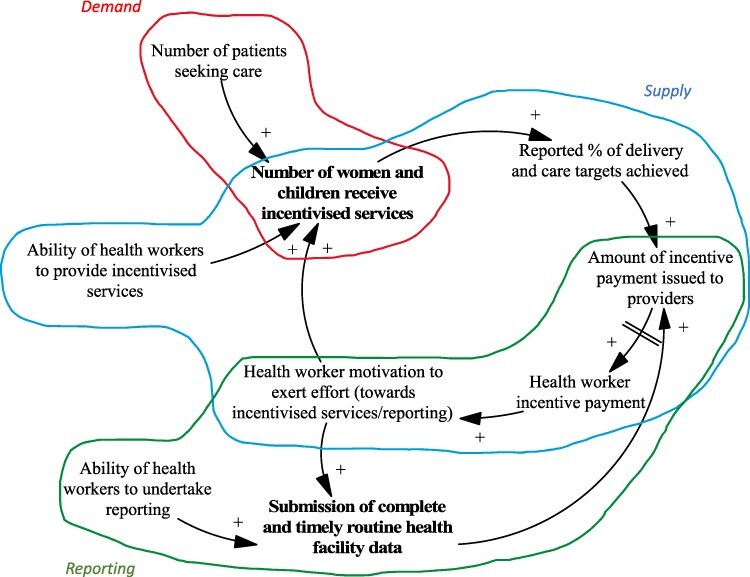
Notes to Box: High-level snapshot of how three smaller diagrams presented in the paper fit together in the larger CLD is given. Three main mechanisms responsible for provider achievement of (or failure to reach) targets during payment for performance programmes are shown here. Mechanisms that result in changes in the supply of services (blue), mechanisms that result in changes in facility reporting (green) and mechanisms that result in changes in demand for services (red). Source: Cassidy *et al.* (2021).

#### Ex-post development

Thematic analysis is a popular choice for extracting information that can then be used for CLD development. Deductive, inductive and blended coding (Skjott Linneberg and Korsgaard, 2019) have been used to analyse primary and secondary sources of data in preparation for CLD development. With the former method, codebooks can be developed using relevant literature, conceptual frameworks and middle range theories (Kwamie *et al.*, 2014; Xu and Mills, 2017) and used to traverse and extract variables, their relationships and linkages to be represented in the CLD. Codebooks can be updated where the researcher identifies new themes during data analysis. Deductive coding provides structure for traversing data from the outset but there is a possibility that new themes and concepts that emerge from the data might be missed.

With inductive coding, codes are derived directly from the data (Renmans *et al.*, 2017; Lembani *et al.*, 2018); codes or categories can be iteratively refined, and data reanalysed. Inductive coding is a suitable choice, where there is a lack of theoretical background to the research topic (Skjott Linneberg and Korsgaard, 2019). In practice, blended coding is often used to harness the strengths of each approach (Elliott, 2018).

Purposive Text Analysis is another option for analysing data and extracting information for CLD development (Kim and Andersen, 2012). This approach involves systematically reviewing key informant transcripts, extracting quotations that describe drivers for behaviour of interest, and extraction of cause-and-effect statements, with diagrams that represent these relationships. Cassidy *et al*. (2021) used this approach to develop their CLD (Box 4) and a method called CLD Combination (Tomoaia-Cotisel, 2018) to systematically merge together key informant CLDs into a single CLD. This approach involves ordering key informant CLDs in order of their ‘complexity’ (number of links, variables and delays). The most complex and second most complex CLD are compared. Additions are made to the most complex CLD where new information about system behaviour is revealed. Key informant CLDs are continually compared to this ‘anchor’ CLD until information from all CLDs are represented in one CLD.

Box 4. **Example of applying Purposive Text Analysis to text**

**(1a) Question:** Are there any strategies being implemented that aim to address these challenges (to provision of quality health services)?

**(1b) Quotation**: ‘Yes, there is strategy done in the district, which is community health fund. We realized that the shortage of equipments and drugs was becoming a common problem which resulted in poor health service delivery [1], the community health fund was established as alternative to solve those problems. So once the government supply insufficient medicine [2] the community health fund money are used to substitute [3/4]’.

**Main argument**: When the Medical Stores Department (autonomous government department that procures and distributes health commodities to facilities, MSD) cannot provide drugs and equipment, facilities must draw on other sources of funding like the community health fund (community-based health insurance scheme) to buy medical commodities.

**(1c) Causal structure:**

**Table:** 

[1]	*Causal variable*	*Relationship*	*Effect variable*
	Stock of drugs/equipment	Increase	Delivery of health services
[2]	*Causal variable*	*Relationship*	*Effect variable*
	MSD issue facility resources	Increase	Stock of drugs/equipment
[3]	*Causal variable*	*Relationship*	*Effect variable*
	MSD issue facility resources	Decrease	Facility use insurance funds to buy resources
[4]	*Causal variable*	*Relationship*	*Effect variable*
	Facility use insurance funds to buy resources	Increase	Stock of drugs/equipment

**Figure:**
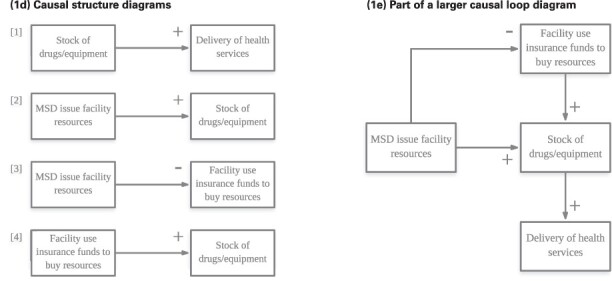
Notes to Box: In the example, the interviewer asked the stakeholder how health providers addressed challenges to the provision of quality health care in their facilities (1a) during a payment for performance programme. Quotations were deemed relevant and extracted if they described events or scenarios that furthered understanding of how stakeholders responded to the programme or demonstrated health system behaviour that facilitated or hindered facilities delivering quality health care (1b). Isolated cause and effect statements, with their associated quotations were extracted from transcripts and stored in an Excel file. The direction of the relationship (positive or negative, see Box 1 for details on interpretation of CLDs) was also noted; in the given example, an increase in the stock of drugs and equipment at facilities resulted in providers being able to deliver health services (1c).At the end of this data extraction process, all cause and effect statements were drawn as simple diagrams with a polarity indicating the direction of the relationship (1d). Each of these simple diagrams were then combined to form a single CLD representative of an individual’s mental model of the system (1e). Stakeholders may not use the same terminology in relaying information; as coding progresses, it becomes easier to standardise variable names assigned to cause and effect statements. Medical Stores Department (MSD).

#### Real time development

For real time development of CLDs, GMB is a popular choice. Scripts are freely available that can help researchers guide GMB sessions (Hovmand *et al.*, 2011), hosted on the Scriptapedia website (Wikibooks Contributors, 2022). There are a range of activities that can be undertaken in GMB sessions depending on the purpose of the workshop; examples include encouraging stakeholders to discuss and list variables they think are driving a system process ‘Variable Elicitation script’ and developing a CLD as a group exercise ‘Initiating and Elaborating a Causal Loop Diagram script’ (Trani *et al.*, 2016; Noubani *et al.*, 2020; Wikibooks contributors, 2022).

A combination of methods can also be used to develop CLDs. For example, researchers may start by developing an initial CLD from secondary data or prospective interviews and then use a GMB workshop to develop a final CLD (Lembani *et al.*, 2018; Jamal *et al.*, 2020). Alternatively, CLDs can be initially developed through GMB sessions before triangulating the results with thematic analysis of subsequent key informant interviews (Zablith *et al.*, 2021). Triangulating the results with data sources or presentation and discussion of the CLD with stakeholders lends weight to the validity of the CLD to represent real health system behaviour (see validation).

### How will I validate the CLD?

The developed CLD needs to be validated to minimize any unconscious bias that may have been introduced by the researcher during development or misinterpretation of data. Stakeholder dialogue is the most popular method to validate CLDs in the LMIC health literature, with illustrative tools provided in Cassidy *et al*. (2021) and Rwashana *et al*. (2014). The decision to approach stakeholders will be dependent on accessibility to stakeholders and the nature of the topic under investigation.

Other examples of validation using primary sources of data include comparison of CLD structure to key informant interview transcripts or the original primary data source used for CLD development (Xu and Mills, 2017; Zablith *et al.*, 2021) and multiple group model building sessions to validate structure (Trani *et al.*, 2016). Secondary sources of data can also be used to validate the CLD, with CLD structure compared to findings in published or grey literature (Alonge *et al.*, 2017; Ahmad *et al.*, 2019), organization reports or policy documents (Paina *et al.*, 2014; Jamal *et al.*, 2020).

It is recommended that for analysis and validation, large CLD structures are broken down into smaller segments (Sterman, 2000b). Cassidy *et al*. (2021) initially split the CLD into three smaller diagrams, related to three broad mechanisms responsible for facility achievement of targets during a payment for performance programme and presented these individual segments to stakeholders for validation. This also allowed presentation of parts of the CLD to stakeholders with knowledge of that sector (rather than presenting the entire CLD for validation). However, initial stakeholder feedback indicated that they were interested in seeing how this smaller segment fed into the wider CLD. The research team felt this was an important issue—in presenting a single segment of the CLD, knowledge of how that segment operates within the wider CLD structure is lost and stakeholders are unable to see the ‘bigger picture’. In future interviews, stakeholders were still asked to comment and provide feedback on one of the three mechanisms, but the mechanism was now highlighted in the wider CLD. An extract of the validation tool used in this study is shown in Box 5, where stakeholders were verbally taken round the CLD to elicit their feedback.

Box 5. Extract of the causal loop diagram validation tool to guide interviews with stakeholders. Original tool adapted from Rwashana *et al.* (2014) and Andersen *et al.* (2012). Source: Cassidy *et al.* (2021), adapted with permission

*The interviewer does not have to explicitly run through these questions while discussing the diagram, can instead probe ‘Does this make sense? Are we missing anything important in this section of the diagram? Is there anything that you feel should be removed in the diagram?’. When an interviewee gives their feedback, it will generally fall into these compartments and help the modeller to go back and make modifications to the diagram:*

● Does this part of the system exist to your knowledge?

● Are appropriate system variables represented? If not, what variables are missing or should be removed?

● Are appropriate in- and outflows represented? If not, what flows are missing or should be removed?

● Is the polarity of in- and outflows accurately represented? If not, what changes would you make? 

● Are appropriate delays in the system represented? If not, what delays are missing or should be removed?

## Conclusion

CLDs are a valuable tool for research or decision making, enabling consideration of problem behaviour, its drivers, and potential health systems policies or interventions as part of a wider, dynamic system. CLDs can identify bottlenecks and leverage points, areas where it would be opportune to intervene to produce optimal system behaviour. They can also be used as direct input to other research tools [e.g. to develop a system dynamics model (Pruyt, 2017)] or complement other research methods [such as realist reviews (Singh *et al.*, 2021) or case studies (Jamal *et al.* 2020)]. Increased familiarity and understanding on how to use systems thinking tools, strengthened science-policy partnerships and dissemination of findings to appropriate audiences are essential to ensure their application to evaluate complex health system behaviour and use of findings (Kwamie *et al.* 2021).

## Reflexivity statement

Article authors consist of four female and two male researchers, with both early career (RC) and senior researchers. Two authors (ARS and PB) are from the article study region.

## References

[R1] Adam T . 2014. Advancing the application of systems thinking in health. *Health Research Policy and Systems* 12: 50. 10.1186/1478-4505-12-50 PMC4245197 25160646

[R2] Adam T , de Savigny D. 2012. Systems thinking for strengthening health systems in LMICs: need for a paradigm shift. *Health Policy and Planning* 27: iv1–3. 23014149 10.1093/heapol/czs084

[R3] Agyepong IA , Kodua A, Adjei S et al. 2012. When “solutions of yesterday become problems of today”: crisis-ridden decision making in a complex adaptive system (CAS) - the additional duty hours allowance in Ghana. Special Issue System Thinking for Health Systems Strengthening LMICs Seizing Opportunity. *Health Policy and Planning* 27: iv20–31. 23014150 10.1093/heapol/czs083

[R4] Ahmad R , Zhu NJ, Lebcir RM et al. 2019. How the health-seeking behaviour of pregnant women affects neonatal outcomes: findings of system dynamics modelling in Pakistan. *BMJ Global Health* 4: e001242. 10.1136/bmjgh-2018-001242 PMC6441297 30997166

[R5] Alonge O , Lin S, Igusa T et al. 2017. Improving health systems performance in low- and middle-income countries: a system dynamics model of the pay-for-performance initiative in Afghanistan. *Health Policy and Planning* 32: 1417–26. 29029075 10.1093/heapol/czx122 PMC5886199

[R6] Andersen DL , Luna-Reyes LF, Diker VG et al. 2012. The disconfirmatory interview as a strategy for the assessment of system dynamics models. *System Dynamics Review* 28: 255–75.

[R7] Barasa EW , Cloete K, Gilson L. 2017. From bouncing back, to nurturing emergence: reframing the concept of resilience in health systems strengthening. *Health Policy and Planning* 32: iii91–4. 29149319 10.1093/heapol/czx118 PMC6626547

[R8] Borghi J , Chalabi Z. 2017. Square peg in a round hole: re-thinking our approach to evaluating health system strengthening in low-income and middle-income countries. *BMJ Global Health* 2: e000406. 10.1136/bmjgh-2017-000406 PMC5656120 29082021

[R9] Cassidy R , Singh NS, Schiratti P-R et al. 2019. Mathematical modelling for health systems research: a systematic review of system dynamics and agent-based models. *BMC Health Services Research* 19: 845. 10.1186/s12913-019-4627-7 PMC6862817 31739783

[R10] Cassidy R , Tomoaia-Cotisel A, Semwanga AR et al. 2021. Understanding the maternal and child health system response to payment for performance in Tanzania using a causal loop diagram approach. *Social Science and Medicine* 285: 114277. 10.1016/j.socscimed.2021.114277 PMC8434440 34343830

[R11] Elliott V . 2018. Thinking about the Coding Process in Qualitative Data Analysis. *The Qualitative Report* 23: 2850–61.doi: 10.46743/2160-3715/2018.3560

[R12] Gates EF . 2016. Making sense of the emerging conversation in evaluation about systems thinking and complexity science. *Evaluation and Program Planning* 59: 62–73. 27591941 10.1016/j.evalprogplan.2016.08.004

[R13] Glenn J , Kamara K, Umar ZA et al. 2020. Applied systems thinking: a viable approach to identify leverage points for accelerating progress towards ending neglected tropical diseases. *Health Research Policy and Systems* 18: 1–15.doi: 10.1186/s12961-020-00570-4. PMC7268457 32493485

[R14] Hovmand P , Rouwette E, Andersen D et al. 2011. Scriptapedia: A handbook of scripts for developing structured group model building sessions. *29th International Conference of the System Dynamics Society* Washington, DC.

[R15] Isee Systems Inc . 2021. STELLA Architect v2.1.4.

[R16] Jamal Z , Alameddine M, Diaconu K et al. 2020. Health system resilience in the face of crisis: analysing the challenges, strategies and capacities for UNRWA in Syria. *Health Policy and Planning* 35: 26–35. 31625558 10.1093/heapol/czz129

[R17] Kanniyan B , Govindarajan PK, Haveri SP et al. 2021. Enactment of system thinking framework for convolution of immunization service governance. *International Journal of Research in Pharmaceutical Sciences* 12: 849–56.

[R18] Kim D . 2000. Part II: Dynamic thinking tools. In: O’Reilly K (ed). *Systems Thinking Tools: A User’s Reference Guide*. Waltham: Pegasus Communications, Inc., 18.

[R19] Kim H , Andersen DF. 2012. Building confidence in causal maps generated from purposive text data: mapping transcripts of the Federal Reserve. *System Dynamics Review* 28: 311–28.

[R20] Kurnianingtyas D , Santosa B, Siswanto N. 2020. A system dynamics for financial strategy model assessment in national health insurance system. *Proceedings of the 2020 2nd International Conference on Management Science and Industrial Engineering*. New York: ACM, 44–8.

[R21] Kwamie A , Ha S, Ghaffar A. 2021. Applied systems thinking: unlocking theory, evidence and practice for health policy and systems research. *Health Policy and Planning* 36: 1715–7. 34131699 10.1093/heapol/czab062 PMC8597965

[R22] Kwamie A , van Dijk H, Agyepong IA. 2014. Advancing the application of systems thinking in health: realist evaluation of the Leadership Development Programme for district manager decision-making in Ghana. *Health Research Policy and Systems* 12: 1–12. 10.1186/1478-4505-12-29 PMC4073809 24935521

[R23] Lembani M , de Pinho H, Delobelle P et al. 2018. Understanding key drivers of performance in the provision of maternal health services in eastern cape, South Africa: a systems analysis using group model building. *BMC Health Services Research* 18: 912. 10.1186/s12913-018-3726-1 PMC6267091 30497460

[R24] Li M , Yu W, Tian W et al. 2019. System dynamics modelling of public health services provided by China CDC to control infectious and endemic diseases in China. *Infection and Drug Resistance* 12: 613–25. 30936725 10.2147/IDR.S185177 PMC6422414

[R25] Lipsitz LA 2012. Understanding health care as a complex ystem. *JAMA* 308: 243. 10.1001/jama.2012.7551 PMC3511782 22797640

[R26] McGill E , Er V, Penney T et al. 2021. Evaluation of public health interventions from a complex systems perspective: a research methods review. *Social Science & Medicine* 272: 113697. 10.1016/j.socscimed.2021.113697 33508655

[R27] Namatovu HK , Semwanga AR. 2020. A systems dynamics approach to understanding the determinants of antenatal care utilization in low-and middle-income countries. *International Journal of System Dynamic Applications* 9: 111–28.

[R28] Nigenda G , González-Robledo LM, Juárez-Ramírez C et al. 2015. Understanding the dynamics of the Seguro Popular de Salud policy implementation in Mexico from a complex adaptive systems perspective. *Implementation Science* 11: 68. 10.1186/s13012-016-0439-x PMC4866010 27177618

[R29] Noubani A , Diaconu K, Ghandour L et al. 2020. A community–based system dynamics approach for understanding factors affecting mental health and health seeking behaviours in Beirut and Beqaa regions of Lebanon. *Globalization and Health* 16: 28. 10.1186/s12992-020-00556-5 PMC7106684 32228648

[R30] Ozawa S , Paina L, Qiu M 2016. Exploring pathways for building trust in vaccination and strengthening health system resilience. *BMC Health Services Research* 16: 639. 10.1186/s12913-016-1867-7 PMC5123384 28185595

[R31] Paina L , Bennett S, Ssengooba F et al. 2014. Advancing the application of systems thinking in health: exploring dual practice and its management in Kampala, Uganda. *Health Research Policy and Systems* 12: 41. 10.1186/1478-4505-12-41 PMC4142472 25134522

[R32] Peters DH 2014. The application of systems thinking in health: why use systems thinking?. *Health Research Policy and Systems* 12: 51. 10.1186/1478-4505-12-51 PMC4245196 25160707

[R33] Pruyt E . 2017. Systems dynamics: a tool for modelling and testing solutions. In: de Savigny D, Blanchet K, Adam T (eds). *Applied Systems Thinking for Health Systems Research: A Methodological Handbook*. London, England: McGraw-Hill Education, pp. 173–94.

[R34] Renmans D , Holvoet N, Criel B. 2017. Combining theory-driven evaluation and causal loop diagramming for opening the ‘Black Box’ of an intervention in the health sector: a case of performance-based financing in Western Uganda. *International Journal of Environmental Research and Public Health* 14: 1–20.doi: 10.3390/ijerph14091007 PMC5615544 28869518

[R35] Rwashana AS , Nakubulwa S, Nakakeeto-Kijjambu M et al. 2014. Advancing the application of systems thinking in health: understanding the dynamics of neonatal mortality in Uganda. *Health Research Policy and Systems* 12: 36. 10.1186/1478-4505-12-36 PMC4134459 25104047

[R36] Rwashana AS , Williams DW, Neema S. 2009. System dynamics approach to immunization healthcare issues in developing countries: a case study of Uganda. *Health Informatics Journal* 15: 95–107. 19474223 10.1177/1460458209102971

[R37] Sarriot E , Morrow M, Langston A et al. 2015. A causal loop analysis of the sustainability of integrated community case management in Rwanda. *Social Science & Medicine* 131: 147–55. 25779620 10.1016/j.socscimed.2015.03.014

[R38] Sharma SR , Matheson A, Lambrick D et al. 2020. The role of tobacco and alcohol use in the interaction of social determinants of non-communicable diseases in Nepal: a systems perspective. *BMC Public Health* 20: 1368. 10.1186/s12889-020-09446-2 PMC7487957 32894104

[R39] Singh NS , Kovacs RJ, Cassidy R et al. 2021. A realist review to assess for whom, under what conditions and how pay for performance programmes work in low- and middle-income countries. *Social Science and Medicine* 270: 113624. 10.1016/j.socscimed.2020.113624 33373774

[R40] Skivington K , Matthews L, Simpson SA. et al. 2021. A new framework for developing and evaluating complex interventions: update of Medical Research Council guidance. *BMJ* 374: n2061. 10.1136/bmj.n2061 PMC8482308 34593508

[R41] Skjott Linneberg M , Korsgaard S. 2019. Coding qualitative data: a synthesis guiding the novice. *Qualitative Research Journal* 19: 259–70.

[R42] Sterman J 2000a. Part 1: Perspective and process. In: *Business Dynamics: Systems Thinking and Modelling for a Complex World*. Illinois, U.S.A.: McGraw-Hill Companies Inc, 22.

[R43] Sterman J . 2000b. Part II: Tools for systems thinking. In: *Business Dynamics: Systems Thinking and Modelling for a Complex World*. Illinois, U.S.A.: McGraw-Hill Companies Inc, 154.

[R0043a] Sterman J . 2000c. Part II: Tools for systems thinking. In: *Business Dynamics: Systems Thinking and Modelling for a Complex World*. Illinois, U.S.A.: McGraw-Hill Companies Inc, 137–41.

[R44] Tomoaia-Cotisel A . 2018. The journey towards the patient-centered medical home: a grounded, dynamic theory of primary care transformation.doi: 10.17037/PUBS.04647856

[R45] Tomoaia-Cotisel A Hyunjung K Allen S et al. 2017. Causal loop diagrams: a tool for visualizing emergent system behaviour. In: de Savigny D, Blanchet K, Adam T. (eds). *Applied Systems Thinking for Health Systems Research: A Methodological Handbook*. London, England: McGraw-Hill Education, pp. 97–114.

[R46] Trani JF , Ballard E, Bakhshi P et al. 2016. Community based system dynamic as an approach for understanding and acting on messy problems: a case study for global mental health intervention in Afghanistan. *Conflict and Health* 10: 1–11 27822297 10.1186/s13031-016-0089-2 PMC5090881

[R47] Valcourt N , Walters J, Javernick‐Will A et al. 2020. Assessing the efficacy of group model building workshops in an applied setting through purposive text analysis. *System Dynamics Review* 36: 135–57.

[R48] Varghese J , Kutty VR, Paina L et al. 2014. Advancing the application of systems thinking in health: understanding the growing complexity governing immunization services in Kerala, India. *Health Research Policy and Systems* 12: 1–12. 10.1186/1478-4505-12-47 PMC4245198 25160531

[R49] Ventana Systems Inc . 2015. Vensim PLE Plus.

[R50] Wikibooks Contributors . 2022. “*Scriptapedia*.” Wikibooks, Free Textb Proj.

[R51] Wilkerson B , Aguiar A, Gkini C et al. 2020. Reflections on adapting group model building scripts into online workshops. *System Dynamics Review* 36: 358–72.

[R52] Xu J , Mills A. 2017. Challenges for gatekeeping: a qualitative systems analysis of a pilot in rural China. *International Journal for Equity in Health* 16: 106. 10.1186/s12939-017-0593-z PMC5493841 28666445

[R53] Yu W , Lv Y, Hu C et al. 2018. Research of an emergency medical system for mass casualty incidents in Shanghai, China: a system dynamics model. *Patient Preference and Adherence* 12: 207–22. 29440876 10.2147/PPA.S155603 PMC5798575

[R54] Zablith N , Diaconu K, Naja F et al. 2021. Dynamics of non-communicable disease prevention, diagnosis and control in Lebanon, a fragile setting. *Conflict and Health* 15: 4. 10.1186/s13031-020-00337-2 PMC7802297 33430916

[R55] Zimmerman L , Lounsbury DW, Rosen CS et al. 2016. Participatory system dynamics modelling: increasing stakeholder engagement and precision to improve implementation planning in systems. *Administration and Policy in Mental Health and Mental Health Services Research* 43: 834–49. 27480546 10.1007/s10488-016-0754-1 PMC8344379

